# An in-depth, exploratory assessment of the implementation of the National Health Information System at a district level hospital in Tanzania

**DOI:** 10.1186/1472-6963-14-91

**Published:** 2014-02-26

**Authors:** Miriam C Wilms, Osman Mbembela, Helen Prytherch, Peter Hellmold, Rainer Kuelker

**Affiliations:** 1 Institute of Public Health, University of Heidelberg, Im Neuenheimer Feld 324, 69120 Heidelberg, Germany; 2 Lugala Lutheran Hospital, P.O. Box 11, Malinyi, Tanzania

**Keywords:** Health information system, District health system, Developing countries

## Abstract

**Background:**

A well functioning Health Information System (HIS) is crucial for effective and efficient health service delivery. In Tanzania there is a national HIS called Mfumo wa Taarifa za Uendeshaji Huduma za Afya (MTUHA). It comprises a guideline/manual, a series of registers for primary data collection and secondary data books where information from the registers is totalled or used for calculations.

**Methods:**

A mix of qualitative methods were used. These included key informant interviews; staff interviews; participant observations; and a retrospective analysis of the hospital’s 2010 MTUHA reporting documents and the hospital’s development plan.

**Results:**

All staff members acknowledged data collection as part of their job responsibilities. However, all had concerns about the accuracy of MTUHA data. Access to training was limited, mathematical capabilities often low, dissemination of MTUHA knowledge within the hospital poor, and a broad understanding of the HIS’s full capabilities lacking.

Whilst data collection for routine services functioned reasonably well, filling of the secondary data tools was unsatisfactory. Internal inconsistencies between the different types of data tools were found. These included duplications, and the collection of data that was not further used. Sixteen of the total 72 forms (22.2%) that make up one of the key secondary data books (Hospital data/MTUHA book 2) could not be completed with the information collected in the primary data books.

Moreover, the hospital made no use of any of the secondary data. The hospital’s main planning document was its development plan. Only 3 of the 22 indicators in this plan were the same as indicators in MTUHA, the information for 9 more was collected by the MTUHA system but figures had to be extracted and recalculated to fit, while for the remaining 10 indicators no use could be made of MTUHA at all.

**Conclusion:**

The HIS in Tanzania is very extensive and it could be advisable to simplify it to the core business of data collection for routine services. Alternatively, the more comprehensive, managerial aspects could be sharpened for each type of facility, with a focus upon the hospital level. In particular, hospital planning documents need to be more closely aligned with MTUHA indicators.

## Background

The World Health Organisation (WHO) defines a *Health Information System* (HIS) as “[…] *a system that integrates data collection, processing, reporting, and use of the information necessary for improving health service effectiveness and efficiency through better management at all levels of health services*” [1]. As such a HIS is far more than a mere ‘data collection’ tool. By transforming data into information that can be practically applied, HIS have the potential to influence the quality of health services and the promotion of health.

A well functioning HIS is crucial for ensuring the effectiveness and efficiency of health care services. “*Good decisions on effective policies, services and behaviour require timely, accurate and relevant information*” [2] p. 1018. In resource-poor settings it is hard to overstate the potential benefit that HIS data can bring to the management of health service provision, and ultimately to the health of populations. This is particularly so as population growth rates and the double-burden of disease are making ever increasing demands of health systems in developing countries [3], whilst the resources available to finance them have stagnated or even decreased [4]. Indeed, it has been commented, *“It is not because countries are poor that they cannot afford good health information; it is because they are poor that they cannot afford to be without it”*[5] p.582.

In Tanzania a national HIS called **M**fumo wa **T**aarifa za **U**endeshaji **H**uduma za **A**fya (MTUHA) was introduced in 1993. It consists of 12 books. These comprise the guideline/ manual (MTUHA book No. 1) and a series of registers for primary data collection (MTUHA books 3–9, 11–12). There are also secondary data books where information from the registers is totalled or used for calculations. At hospital level the primary data collection (filling of registers) takes place at the respective duty station (ward, theatre, OPD) during the clinical routine.

The primary data books provided by MTUHA are comprehensive in scope. They cover all the data categories foreseen by WHO including: ‘surveillance systems’, ‘routine service reporting’, ‘administrative reporting’ and ‘vital registration’. A major focus lies on maternal and child health - with data for this collected in four out of the nine registers, as well as the collection of more general information from outreach work in a ‘community book’, a ‘ledger book’ to monitor the flow of drugs and supplies, an ‘OPD-register’, a ‘dental register’ and a ‘diarrhoea-treatment-corner’ for routine clinical data collection.

MTUHA does not provide an admission book or a theatre registration book. However it is recommended by the MTUHA guidelines/manual that such books be designed and put into use at facility level.

The secondary data books at hospital level are MTUHA book 2 and MTUHA book 10. The latter is also known as the Hospital Report. Book 2 summarizes data from the primary books and comprises different tables. According to the MTUHA guideline/manual the purpose of book 2 is to facilitate completion of book 10. These two secondary data books comprise forms that need to be filled out on a monthly, quarterly or yearly basis, and are used for reporting to the next level in the service structure.

The levels of service structure in Tanzania include dispensaries that serve several villages (ward level), health centres serving the divisional level, hospitals at district and regional levels and at the central level the Ministry of Health [6].

**Figure 1:**
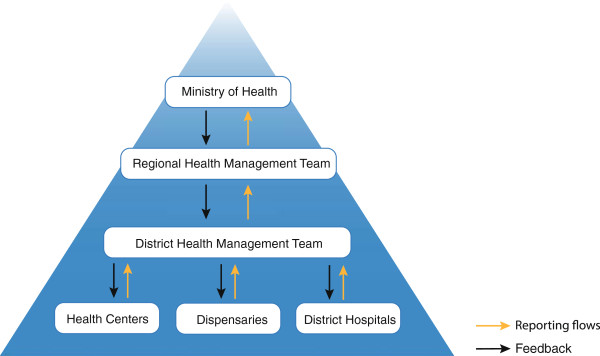
MTUHA reporting and feedback flows.

Figure 1 shows how data for reporting and feedback should flow between the different administrative and service levels.

HIS show poor performance in many developing countries and challenges at different levels have been acknowledged [4,5,7]. In many decentralized health systems the district level has been found to be particularly weak [8], despite being the essential service structure for the provision and coordination of hospital level services. In the case of Tanzania previous studies have shown shortcomings of MTUHA at various levels [9,10]. The Tanzanian Ministry of Health acknowledges that (in the national HIS) “(…) *there are weaknesses: data from health facilities are not always complete or not reliable. Often data collection is delayed. Feedback to collecting facilities, particularly from the district level is practically nonexistent”*[11].

The recognition of the central importance of a functioning HIS within health systems has led to the ongoing process of HIS-strengthening and restructuring that is widely known as HIS-reform. Global developments such as the need for surveillance systems to deal with pandemics and stricter monitoring for global health initiatives have intensified the importance of HIS-reform in recent years [2]. Nevertheless the literature on the subject only highlights the most common HIS shortcomings in a very general manner. There is a dearth of literature providing a detailed exploration of HIS implementation at hospital level. Policy-makers in Tanzania have called for more research in this area to enrich the ongoing process of reforming MTUHA [12].

This paper provides an in-depth, descriptive and exploratory assessment of the functioning of MTUHA at one district level hospital. This is achieved through an assessment of MTUHA use and an exploration of hospital staff views and experiences. Based upon this, practical steps for the improvement and strengthening of MTUHA at hospital level are proposed.

## Methods

This exploratory assessment was both cross-sectional and descriptive in nature. Its aim was to establish the functionality of the MTUHA system at hospital level. The objectives were to assess how well the data collection, data analysis/reporting and use of information processes worked *within the hospital* and where challenges occurred. A mix of qualitative methodologies were used to assess these three stages of data handling in MTUHA. Using a mix of methodologies was considered advantageous as it allowed for the triangulation of results for consolidation.

The methods comprised key informant interviews; staff interviews; participant observations; and a retrospective document review of the hospital’s 2010 reporting books, its development plan and the information collected at the hospital by the National AIDS Control Program (NACP). Each method is described in detail below:

### Key informant interviews

The key informants interviews made use of a semi-structured design to obtain a deeper understanding of the context in which MTUHA was embedded and experiences with MTUHA within the hospital. Key informants were defined as persons with responsibilities that extended beyond the mere collection or reporting of HIS data at the hospital. They were selected using a judgement approach [13]. Four individuals were thus identified and all of them accepted the request to be interviewed. They included: the Medical Officer in-charge who is responsible for the completion of the MTUHA quarterly and yearly report before they are passed on to the District Level; the Country Coordinator of a European NGO that technically assists the hospital; the Head Nurse who compiles the data from different departments and fills out the MTUHA books 2 and 10 (Hospital data book and Hospital report) and the Headmaster of the attached Nursing School who teaches MTUHA data collection.

### Staff interviews

Interviews were used to explore the experiences of hospital staff involved in, and responsible for, MTUHA data collection. The staff interviews were structured, mainly using closed questions to produce results that could be compared within this group of interviewees. The interview guideline firstly addressed the respondents profile and educational background before exploring their knowledge of, and general attitude to, MTUHA, their experiences with MTUHA, including problems encountered, feedback received, and their suggestions for improvement. It was developed in English before being translated into Kiswahili and pretested before use. During these interviews, once the respondents had given their spontaneous answer, probing was used to address any hitherto defined aspects that had remained unaddressed. All 18 of the non-auxiliary clinical staff (assistant medical officers, clinical officers, A-level and B-level nurses, midwives) were approached and 17 agreed to take part. They included the in-charge and additional staff members from the following departments: Reproductive & Child Health Clinic, Counselling and Testing Clinic (CTC), Out-Patients, Maternity, Adult Ward, Paediatric Ward, Theatre and Radiology. The interviews were all conducted by the first author in English, German or Kiswahili and notes were taken. On average the interviews took 20 min.

### Participant observation

Participant Observation is a method in which the researcher takes on a certain role in the community under study to attain further knowledge about details such as work relations, hierarchies and time management or hidden details such as taboos, rivalries, etc. [14]. In this assessment it was used to gain a deeper understanding of the organisational structures involved in MTUHA at the hospital, to determine who interacts with whom in relation to MTUHA, to establish how much time is spent on MTUHA-related tasks and what the practical obstacles hinder its smooth implementation tasks [15]. This assessment provided for 75 days of observation, a timeframe which others have used for such explorations and found to be sufficient [16].

During participant observation, the first author had a role as an observing medical doctor during clinical work, including participation in daily morning meetings, following ward rounds, observing patients’ consultation in the OPD and observing surgical work in the theatre. During these activities the researcher was involved in the data collection for MTUHA by; (i) assisting the use of MTUHA Book 5 at the OPD, (ii) assisting the use of the Major and Minor theatre book, and indirectly by (iii) discussing patients diagnosis and treatments. She also assisted the Medical Officer in charge in the compilation of the information needed for the NGO’s annual report.

### Retrospective document review

This review was used to assess the data collection within MTUHA for completeness, mathematical correctness, consistency and plausibility Since the hospital had undergone a change in leadership in 2009 and the current medical officer in-charge had only joined in May 2009, the data that was reviewed was restricted to the year 2010.

All available MTUHA books were collected (those missing were noted), and assessed by checking the following details:

Primary Data books (MTUHA books 3–9,11-12):

– Registers were inspected to see if they had been filled according to the MTUHA Guidelines/manual (book 1)

– Single entries in registers were counted and totalled according to MTUHA guidelines. These totals were compared to those documented in the MTUHA books to establish if any counting errors had taken place

– The totals were compared to the entries in the Health Facility Data Book (book 2)

Secondary Data books (MTUHA books 2 and 10)

– eForms were checked for completeness; it was noted if they were only partially or not at all filled out.

– Indicators were assessed as to whether they seemed reasonable in the context of the hospital.

– Indicators calculated in MTUHA were compared to the indicators recalculated for the annual report of the supporting NGO.

The assessment focused on MTUHA as this was our main subject of interest. However to be able to demonstrate the workload of data collection, as well as the limits of MTUHA all additional data collection performed at the hospital was also documented, reviewed and referred to. The findings from the various methods were analysed separately and later consolidated for the final interpretation and reporting of results [17].

The assessment was conducted over a period of two months at a rural faith-based hospital at district level in South-West Tanzania. Permission was obtained from the Tanzanian Commission for Science and Technology (no. 2012-67-NA-2011-70) and from the management of the hospital concerned. Ethical clearance was granted by the National Institute for Medical Research in Tanzania. The purpose of the interviews was explained to all key informants and hospital staff and verbal consent attained. The results of the staff interviews were kept anonymous to maintain as much confidentiality as possible.

### Limitations

The choice of a faith-based hospital rather than a public district hospital for this assessment was arguably a limitation. However the opportunity provided for such an in-depth assessment was considered important enough to overrule this concern. Public-private partnership arrangements in Tanzania dictate that such a hospital has the same obligation to contribute to MTUHA as a public one. It is possible that staff at such a hospital might have had less access to MTUHA training than staff in a government district hospital, as has been found to be the case of other training areas [18]. Faith-based hospitals do not have any formal responsibilities for undertaking outreach activities in the district or supervising other health facilities, but these functions were not the subject of this assessment.

The timing of the assessment might have been a limitation especially for the participant observation. The one-off nature of the exercise also meant that seasonal effects upon the number of patients (e.g. dry season versus rainy season) could not be observed.

The assessment focused exclusively on the use of MTUHA at hospital level in keeping with the intention to provide an in-depth and detailed picture. MTUHA is also used at other levels which were not examined here. Moreover, when it came to exploring the use made of the MTUHA information e.g. at the district level, the assessment is unlikely to show the entire picture. Further research could usefully be undertaken in these areas.

Finally, the findings may resonate amongst those familiar with the setting and generate a broader recognition [19]. Indeed, they may be transferrable to other similar, rural hospital settings in Tanzania. However, they cannot be generalised in a statistical sense.

## Results

The results from all of the methods used are presented together according to the three main processes within the HIS – data collection, data analysis/reporting and data use.

### Data collection

The staff interviews showed the hospital staffs’ general attitude towards data collection was positive with all interviewees acknowledging the importance of a well functioning HIS. Furthermore, all staff members acknowledged data collection as part of their job responsibilities.

However, the participant observations found that there was no medical record department where all the books from recent years were collected and stored as required by the MTUHA guidelines/manual (book 1). Instead the books were stored at each department and when staff members were asked for them they often had to refer to the department manager to establish their whereabouts.

The retrospective document review found that the MTUHA Book 3 (Community Book) and Book 9 (Diarrhoea Treatment Corner) had not been used for the year 2010. At the maternity the MTUHA book 12 could not be found from January until May 2010. Therefore only the data from June to December was available. These books were generally well filled with complete data provided for an average of 9 patients per day. However, during participant observation several neonatal deaths were witnessed. This stood at odds with the generally low number of such deaths that were reported.

In addition to MTUHA, further registers were in use to collect data for the Prevention of Mother to Child Transmission (PMTCT) program and the Provider Initiated Testing and Counselling (PITC) program. The document review showed there to be some confusion between the two programs as women who came in labour for delivery were occasionally entered in the PITC register instead of the PMTCT register.

In the Reproductive and Child Health Clinic in total three MTUHA books and three additional registers (PMTCT, PITC and Expanded Program of Immunisation (EPI)) should be filled. In the staff interview a very heavy reporting workload was described. Despite this, data collection tools were generally observed to be correctly used in this department and the document review confirmed this to be the case for the 20–80 entries per day.

As recommended by MTUHA there was an admission form on each ward that included the patients’ name and age as well as the initial diagnosis, final diagnosis and treatment. On the wards the participant observation found that the admission books were filled out tediously. Participant observation showed, and the staff interviews confirmed, that there were an average of 5 admittances or discharges each day all of which were documented. The document review revealed that there was generally just one diagnosis given for each patient suggesting that some diagnosis get lost. The initial diagnosis and final diagnosis were often the same and were not always consistent with the treatment, e.g. initial and final diagnosis: ‘urinary tract infection’, treatment: ‘appendectomy’.

The document review showed that the accuracy of the monthly summaries was poor due to two main problems: firstly, diagnoses were documented inconsistently; secondly, counting mistakes were frequently found when entries had been totaled.

The key informant interviews gave some more background on why the entry of the diagnosis presented difficulties: To standardise the diagnosis MTUHA provides a ‘classification of disease’. This is a list with 41 diagnoses that are partly specific such as ‘malaria, all types’, and partly very general such as ‘dental’ or ‘skin infection’. It became apparent that this was perceived to be a major weakness as, *“it is not comparable to the International classification of disease (ICD)”*, moreover, *“some of the categories are simply outdated such as the terms ‘neurosis’ and ‘psychosis’.* Also anaemia is *“included as a disease but it is rather a symptom that can exist in many diseases so that cases like malaria or hookworm infection might be hidden under the term anaemia*””.

With reference to the frequent calculation mistakes found in the monthly tally sheets, the key informants highlighted the staffs’ poor mathematical skills to be a major challenge to the correct use of MTUHA. In addition, they drew repeated attention to the poor dissemination of knowledge between those who were well informed about MTUTHA and other hospital staff.

Beyond these issues, a systematic error was found in the way the length of stay was documented: it always included the day of admission and the day of discharge (instead of just the nights of stay as outlined in the MTUHA guidelines/manual) and therefore one day too many was recorded for all patients.

The OPD registers (book 5) were observed to be available. Summing the entries in the OPD register (book 5) from the year 2010 gave a total of 6,169 OPD patients. That would amount to an average of about 17 patients a day. Despite mention of seasonal variation in the staff interviews the document review revealed that the monthly totals remained similar throughout the year. During participant observation the register was seen to be filled for most patients. However key informants as well as the staff repeatedly said that data collection at OPD was a problem and that too few patients were recorded. When pushed for an explanation it emerged that if the clinical officer attending at the OPD wanted an Assistant Medical Officer or Medical Officer to see a patient then they sent the patient to wait outside the theatre. No documentation at all was filled out for such patients. There could reportedly be 20–40 such patients per day all of whom went completely unrecorded.

When reviewing the hospital’s MTUHA documents it was unclear whether documents were not filled out because information was not available or there had been no such cases. If a service was not available, or if a question in the MTUHA book was not applicable ‘0’ or ‘negative’ was documented. Taken out of context this could be misleading. E.g. there were ‘0’ forceps extractions, there were ‘0’ patients receiving physiotherapy, the third sputum test for acid-fast bacteria (test for tuberculosis) was always negative. This was because these services were simply not provided by the hospital.

Data collection at the Care and Treatment Clinic (CTC) was separate from the MTUHA system, having been designed and introduced later by the National AIDS Control Program (NACP). The NACP database is a computer based information system with a multitude of HIV/AIDS related indicators. The documentation and analysis of the data was well understood and performed by the staff of the CTC. Extensive training was reported to have taken place. Monthly, quarterly and yearly reports could easily be generated through the computer system.

The interviews provided further information that help to explain some of these findings. Even amongst the key informants a strong knowledge of MTUHA was lacking. Three of them reported not having read the MTUHA guidelines/manual or undergone any training in MTUHA. The head nurse who collected the totalled registers from the different departments every month described difficulties in receiving complete, accurate and timely information. *“The quarterly report for the District Health Management Team (DHMT) is due on the 3rd of the following month, but when I go to the different departments at the end of the quarter to collect the reports often the summaries of each department have not been finalised. That leaves very little time for me to fill the quarterly report.”* Her knowledge of how MTUHA should work was good as she could give a detailed overview of the different books and had read the MTUHA guidelines/manual, despite never having received any training on MTUHA or data collection in general.

The staff interviews showed there to be some who could not describe MTUHA at all, whilst most could not define it further than “the books to enter data”. Indeed, only one could give a complete and comprehensive definition. The majority were unable to state how many MTUHA books there were. Over half the respondents reported never having had any training in MTUHA. Just two reported having read the MTUHA guidelines/manual. All of the key informants and most of the other staff interviewed were of the opinion that the data collected by MTUHA was not accurate.

The respondents gave the following reasons for suboptimal use of MTUHA at the hospital: time constraints; lack of clarity as to whether return visits should be entered; how diagnoses were often unclear resulting in forms not being completed; lack of motivation to fill the forms all the time; failure to understand the reasoning behind some of the forms; language difficulties as some of the forms are in English and not Kiswahili; information not always available – e.g. patients not able to give their age; differing formats for the quarterly and yearly reports. Some resented that there was no monetary incentive for data collection as was reported to be the case for the HIV/AIDS reporting system run by the National AIDS Control Program (NACP).

### Data analysis and reporting

#### Use of the secondary data tools

Two inconsistencies between the hospital data book (MTUHA book 2) and the Hospital Report (book 10) were observed that impeded efficient use. Firstly, of the 20 indicators that had to be calculated for MTUHA books 2 and 10, two were duplicated and appeared in both books. Secondly, the data collected for the indicators in book 2 was found not to be included in any of the reports that had to be submitted to the district level.

Overall, MTUHA book 2 was largely incomplete. That is, it lacked information foreseen for collection in the MTUHA guideline/manual. Of the 72 forms 27 (37.5%) were filled completely, 19 (26.4%) partially (at least one section not filled) and 26 (36.1%) were not filled at all. Of the 26 forms that had not been filled 11 of them belonged to routine service reporting, whist the others all had to do with administrative service reporting. Indeed, during the document review it became apparent that 16 of the 72 forms (22.2%) making up book 2 could not be completed from the data collected in the primary data books or associated books such as the admissions or theatre books. The key informant interviews revealed that whilst some of this information did exist in the hospital – e.g. about human resources or financial management – only certain, generally non-clinical staff had access to it.

The Hospital Report (book 10) showed similar, significant deficiencies. The extent to which the different forms that make up this report had been filled out is shown in Table 1.

**Table 1:** Completeness of forms in MTUHA book 10 for year 2010

**No**	Complete*	**Incomplete**	**Not filled**
F001		X	
F002		X	
F003		X	
F004 1st quarter		X	
F004 2nd quarter		X	
F004 3rd quarter		X	
F004 4th quarter		X	
F005		X	
F006			X
F008			X
F009			X

*Complete meant that all sections were filled out. Incomplete meant that at least one section was not filled.

Of the 11 forms 8 were filled incompletely and 3 were not filled at all. However, Part 7 of F004 which summarises the routine service reporting was completed for all quarterly reports.

Some of the figures in book 10 were not accurate or consistent with the data collected in the primary data books. Table 2 gives an example of inconsistencies between the figures attained through the OPD register (MTUHA book 5) and the figures noted in book 10.

**Table 2:** Entries by OPD MTUHA book 5 as counted and as reported

	**Counted by the first author (using the data from MTUHA book 5)**	**Figure given in MTUHA book 10**	**Figure given in hospital yearly report**
1st quarter	2000	2591	n.a.
2nd quarter	1345	2534	n.a.
3rd quarter	1130	3780	n.a.
4th quarter	1694	2253	n.a.
Total	6169	11049*	11049

*11049 is derived from the above numbers with a calculation error. The actual number would be 11158.

At the time of research the head nurse was responsible for filling book 2 and 10 and forwarding the relevant parts to the district level. She mentioned that even when she received the data from the departments it was hard for her to calculate the indicators in book 2 and to eventually fill the reports for book 10. This could be supported during participant observation. Moreover, she complained that she tried to get the data from each department by the third of each month but most of the time it came late so the report submission was also often delayed. She reported that very late submission was noticed and followed up by the district health management team.

The review of the Hospital Report showed that in its Quarterly Report on routine data collection (Form F004) summarizes the clinical data divided into 11 subsections spread over 4 pages. 45 figures collected in the primary data tools have to be transferred to this form and then, using this data, 9 indicators have to be calculated. Of these 9 indicators, 7 are population based indicators and 2 are facility based indicators. The details of form F004 are described in Table 3.

**Table 3:** The different parts of F004 and their functions

**Part**	**Information**	**Type of Data/Comment**
1	Documentation of management issues:	No number. qualitative
	− Date of quarterly HMT meeting	
	− Date of quarterly CHMT meeting	
	− Date of meeting with village community committee	
2	Documentation of stock-out for a selected number of drugs by days per month that drug is not available	Absolute number: 24 different drugs listed, including Chloroquine tabs/injection/ syrup*
3	Documentation of stock-out for a selected number of drugs by days per month that drug is not available	Absolute number: part repetition of part 2, 8 different drugs listed, including Chloroquine tabs/injection/ syrup*
4	Control of cold-chain for polio vaccine:	Absolute numbers
	− No of vials discarded due to cold chain failure	
	− No of bottles received	
5	Delivery of “drug kit”:	Absolute number
	− No of days the “drug kit” is received late	
6	Outreach data from “the village”:	Absolute numbers: “the village” is not defined therefore the catchment population for the outreach activity is unknown
	− No of children <5 that died	
	− No of women in fertile age	
	− No of newborn death	
	− No of cases with neonatal tetanus	
	− No of children weight	
	− No of children underweight	
7	Health service data:	Absolute numbers: to be taken from the primary data books or the summary of them in MTUHA book 2. These absolute numbers are then used in part 8 (below) to calculate indicators.
	− No of OPD patients	
	− No of dental clinic patients	
	− No of recurrent visit at dental clinic for complications	
	− No of newly enrolled patients at RCH clinic	
	− No of <1 year olds receiving tetanus vaccination	
	“Diarrhoea treatment corner” (DTC) data:	
	− No of patients treated in DTC	
	− No of dehydrated patients treated in DTC	
	ANC-Clinic:	
	− No of pregnant women enrolled	
	− No of pregnant women tested for syphilis	
	− No of pregnant women positive for syphilis No of pregnant women receiving 2–5 dosages of tetanus vaccination	
	Maternity:	
	− No of facility based deliveries and deliveries on the way	
	− No of deliveries by traditional birth attendant	
	− No of all deliveries	
	Vaccinations:	
	− No of < 1 year olds vaccinated for BCG	
	− No of < 1 year olds vaccinated for DPT	
	− No of < 1 year olds vaccinated for DPT3	
	− No of < 1 year olds vaccinated for polio	
	− No of < 1 year olds vaccinated for measles	
	− No of children weighed at time of measles vaccination	
	− No of children underweight at time of measles vaccination	
	Supplements:	
	− No of postnatal children that received vitamin A supplement	
	− No of children that received vitamin A supplement at measles vaccination	
	Family planning:	
	− No of women on family planning	
	− No of new clients on family planning	
8	**Workload: No of OPD patients per month/working days available in OPD per months**	Facility based indicator
	ANC coverage: Clients coming to ANC/total number of < 1 year olds per year**	Population based indicator
	**Tetanus coverage in pregnancy: ANC clients that received 2–5 dosages of tetanus vaccination/total of ANC client**	Facility based indicator
	Sum of deliveries performed at the facility or by birth attendance/total number of < 1 year olds per year**	Population based indicator
	RCH coverage: No of children enrolled at RCH/total number of < 1 year olds per year**	Population based indicator
	DPT3 vaccination coverage: No of < 1 year olds who received DPT3/total number of < 1 year olds per year**	Population based indicator
	Measles vaccination coverage: No of < 1 year olds that received the measles vaccination/total number of < 1 year olds per year**	Population based indicator
	Malnutrition prevalence: No of children that were underweight at time of Measles vaccination/total number of < 1 year olds per year**	Population based indicator
	Family planning coverage: No of new clients on family planning/total of women in fertile age***	Population based indicator
9	List of additional concerns: complaints not previously recorded to be noted	n.a.
10	Notifiable disease statistic: No of clients with the 11 notifiable diseases (list provided by Tanzanian MOH)	Absolute numbers
11	List of development activities planned by the administration	n.a.

*Chloroquine was banned from use Tanzania in 2000 [20].

**this denominator is given by the DHMT. It is the estimated total of <1 year olds for the district.

***this denominator is given by the DHMT. It is the estimated total of women in fertile age (15–49) for the district.

Indicators are written in bold letters.

Table 3 shows that most of the central indicators are population based indicators. For a well functioning district health system – of which such a hospital forms a key component – the catchment population must be known. It was found to be accepted practice that the district health management team supplied a figure for the hospital catchment population at the beginning of each year. The figure given for the year 2010 was 106,714. None of the key informants were able to shed any light on how this figure had been calculated. The head nurse stated that the figure had remained unchanged for several years. Using data from the last Census in 2002, and taking staff experience of patient origin, as well as geographical considerations regarding accessibility, into account we estimated the catchment population of the hospital to be about 200,000.

This difficulty permeates the design of MTUHA. For example, Table 3 also shows that the vaccination coverage has to be calculated. The numerator was attained by counting the number of children vaccinated at the RCH-Clinic (facility-based). The denominator (population to be vaccinated) was another population based figure that the district had supplied. For the year 2010 the figure given was 76 under 1 year olds. This is a significant underestimation if either the catchment population of 106,714 or the more reliable 200,000 is taken as the size of the catchment population. This miscalculation is the likely explanation for the observation that the RCH-clinic recorded a vaccination coverage for at least two dosages of tetanus of 100%, whilst at the same time the nurses reported that the vaccines were usually out of stock by the middle of each month. This information could be supported by the data from the ledger book for vaccines.

The Yearly Report (Form F005) is the second essential document within MTUHA book 10, besides the quarterly report (form F004). It consists of eight pages that in essence provide a summary of the data from the quarterly reports (form F004). Here there are a further three indicators that need to be calculated and again, one of them is a populated based indicator concerned with the percentage of people in the catchment area that use OPD services.

### Feedback mechanisms

The staff interviews showed that only the department managers had ever received any feedback on the data submitted. In most cases the context in which the feedback was given had nothing to do with the MTUHA system – coming, for example, from auditors at the pharmacy, through the supporting NGO or from staff working in the National TB program. The staff mainly saw the Medical Officer In-charge or the Head Nurse as being responsible for delivering feedback. However, the former stated that no feedback was ever received from the district or above: *“I sign the MTUHA documents when the Head Nurse gives them to me. After that I never hear anything about them again”.* MTUHA was also not reported to be a focus of supportive supervision – even when it was carried out within some vertical programs. One staff member commented, *“When the district leprosy coordinator came for supportive supervision I asked him how high the prevalence of leprosy in the district is. He could not tell me as he did not know it himself.”*

### Data use

#### Use of information within the hospital

None of the data generated by MTUHA book 2 or 10 was directly used within the hospital.

Neither the key informants nor the staff that were interviewed were aware that MTUHA had also been designed for use as a hospital management tool. Only one staff member described MTUTHA as being anything more than a routine system for data collection. In particular, the hospital management had poor knowledge of the MTUHA books. During participant observation it became clear that MTUHA was not discussed in hospital management meetings.

The key hospital planning document was its development plan. This was referred to in hospital management meetings, as well as meetings with the supporting NGO and any other visitors. The hospital development plan used indicators chosen by the funding NGO. It was stated by the country coordinator of that NGO that this selection had been made in line with the specific programs that were funded so that feedback could be given to the donors.

Table 4 shows that of the 22 indicators in the hospital development plan only 3 were identical with indicators from MTUHA, for 9 the information was available in MTUHA, for 4 the information was available in the NACP data base, while for the remaining 6 MTUHA did not supply the information at all. During participant observation it became clear that MTUHA was not referred to when the indicators for the hospital development plan were calculated.

**Table 4:** Ability of MTUHA to generate indicators/information for the hospital development plan

**Indicators used in hospital development plan**	**Indicator found in MTUHA**	**Information available in MTUHA**	**Can/How could MTUHA be used for greater alignment**
Stillbirth/1000 live birth at hospital	No	Yes MTUHA book 10, F004	Use perinatal mortality (MTUHA book 2, Table 23 D)
Hospital based mortality of children <5 (<5 death/total live birth at hospital)	No	Yes MTUHA book 2	No
<5 death due to acute respiratory infection (ARI)/total <5 admitted due to ARI	No	Yes MTUHA book 2	No
<5 death due to diarrhoea/total <5 admitted due to diarrhoea	No	Yes MTUHA book 2	No
<5 death due to malaria/total <5 admitted due to malaria	No	Yes MTUHA book 2	No
Facility-based maternal death ratio: maternal death/100000 live birth at hospital	Yes	*(Yes as noted in previous column)*	n.a.
	MTUHA book 2, Table 23C		
Proportion of C/S/total deliveries at hospital in%	No	Yes MTUHA book 10, F004	No
Perinatal mortality rate: perinatal death/1000 deliveries at hospital	Yes	*(Yes as noted in previous column)*	n.a.
	MTUHA book 2, Table 23 D		
Proportion of ANC clients tested for HIV: ANC clients tested/total of first visits at ANC	No	Yes MTUHA book 10, F004	No
Proportion of children <5 enrolled at RCH from all children <5 of catchment area	No	No	Use RCH coverage: No of children enrolled at RCH/total number of < 1 year olds per year
			(MTUHA book 10, F004, part 8)
Pregnant women enrolled at RCH from all pregnant women in catchment area	Yes	*(Yes as noted in previous column)*	n.a.
	MTUHA book 10, F004		
Proportion of person participating in VCT from all couples in catchment area*	No*	No	No
Proportion of couples participating in VCT from all couples in catchment area*	No*	No	No
ART retention rate (number of Patients on ART by end of the year/total of Patients ever treated for HIV since 2006)	No*	No	No
PMTCT enrolment coverage (number of pregnant women on ARV therapy/number of pregnant woman tested positive for HIV)	No*	No	No
Earnings/Expanses	No	No	No
Earnings from patients-fees/total earnings	No	No	No
Average expenses per patient	No	No	No
Bed-utilisation-rate	No	No	No
Average length of stay	No	No	No
Proportion of staff available from staff eligible	No	Yes MTUHA book 2	No
Proportion of staff having had training from all staff	No	Yes MTUHA book 2	No

*this data can be found in the National AIDS Control Program (NACP) data base.

The supporting NGO took the opportunity of this assessment to align the indicators they used for monitoring and evaluation with the HIV/AIDS indicators collected routinely by the NACP. The last column of Table 4 suggests how MTUHA indicators could be used in the hospital development plan and, as such, could form the basis of a similar alignment process for other areas.

## Discussion

### Data collection

MTUHA was designed to fit the comprehensive definition of an HIS as provided by WHO. It is more than simply a tool for collecting routine service data. It is a management tool that also covers administrative issues such as human resources, financing of health services, equipment procurement and maintenance. Our findings suggest that it is precisely this comprehensiveness that poses a problem for the data collection in MTUHA.

The data collection tool, namely the MTUHA books, were shown to be partly inconsistent: A significant amount of the secondary data demanded could not be generated by the primary data books. This was especially true for the administrative data. Even if this data existed in the hospital the staff that was responsible for MTUHA did not have access to it. Rather it is collected and monitored by non-clinical staff. In a setting where hierarchies are strong and differences in staff trainings are a cause of snobbery [21], the free sharing of such information is unlikely to take place without managerial intervention.

The failure to update MTUHA is a further limitation, and one that could be quite easily addressed. Whilst a strategic decision appears to have been taken not to incorporate HIV/AIDS data collection as the epidemic unfolded in Tanzania, the list of diseases in MTUHA could be screened and aligned with the current burden of disease. Given the rise of non-communicable disease in Tanzania [22], these could be taken up. Obsolete terms could be replaced and current drug regimens inserted. This would have the advantage of making MTUHA appear more relevant and responsive and make it more likely to be referred to.

The key informants and staff were found to be sceptical about the accuracy of the data collected by MTUHA. This view corroborates the results of other studies [7,9]. However, this broad perception of inaccuracy requires a more differentiated dissection. It is possible that it is largely based on a generalisation of the shortcomings of the administrative data collection. In fact, this assessment showed that the primary collection of routine service data at the hospital had acceptable levels of both accuracy and completeness. This was especially so in the maternity and RCH Departments, for HIV/AIDS and on the wards. With regards to RCH and HIV/AIDS this may be because vertical programs in these area have paid more attention to, and expressed an interest in, the figures reported. The design of MTUHA in this regard was consistent and well understood by the staff. As the current needs for information about health indicators by the Tanzanian Ministry of Health and development partners are mainly focused on routine service data collection, the potential of MTUHA as a routine service data collection tool needs to be highlighted to encourage its use as such.

The assessment found that an in-depth understanding of MTUHA’s potential as a management tool was not present. As a result it was not used as such. Moreover, MTUHA is used at all levels of the health system and it seems ambitious for it to try and cover all the different management issues facing facilities ranging from dispensaries and health centres up to regional, referral hospitals. If the Ministry of Health seeks to maintain MTUHA’s focus upon management aspects the question must be asked whether the guidelines and data collection should not be targeted to each separate level. By simplifying the management component of MTUHA to a basic minimum, the routine service data collection, which is clearly accepted by staff, could be strengthened.

Besides these challenges associated with the design of MTUHA and essentially the definition of a HIS, practical obstacles to the successful implementation of MTUHA were also revealed: This hospital, like the whole health sector in Tanzania, faces a severe human resource crisis with only 49,5% of the foreseen posts filled. The few staff that are present already face competing demands on their time and data collection increases their workload still further. The human resources situation has also led to staff with little formal education filling posts according to their practical ability [21]. Their knowledge of data collection might not be sufficient.

Even though MTUHA is covered in the pre-service training of health staff and the teaching staff involved had a good general knowledge of it, this does not seem to have translated into flawless use of MTUHA at the hospital. Reasons for this are likely to include how MTUHA is taught – for example, whether it is simply conveyed as a data collection tool, whether problem-based learning is used and whether newly trained staff are able to implement what they have learned. This is an area that requires further investigation. A general failure in the sharing and dissemination of information about MTUHA within the hospital emerges. This is likely to be a side-effect of the limited understanding of MTUHA’s potential as a whole.

The impact of the educational sector on the health sector has also to be considered, as the general mathematical knowledge amongst some of the staff was observed to be poor. Specialised short courses on MTUHA are not likely to yield satisfying results if the basic educational level of the staff is not taken into account. It is also a weakness that given the broad conceptualization of MTUHA, non-clinical, administrative staff are not foreseen as targets of the current MTUHA trainings.

### Data analysis and reporting

The secondary data books deliver a total of 20 indicators from MTUHA book 2 and MTUHA book 10. This assessment identified several problems associated with these indicators:

Firstly, seven out of 20 indicators are population-based indicators that use the respective part of the catchment population as their denominator. This calculation should not be undertaken at the facility level but – as foreseen – by the district, regional and national level, using the absolute numbers submitted from each health facility in the respective area. In this assessment the figures supplied by the district were clearly inaccurate. The ramifications of this inaccuracy were clearly shown at the hospital’s RCH-Clinic where the vaccination coverage for at least two dosages of Tetanus – based upon a figure that was clearly incorrect- reached 100% in the year 2010. This result was misleading in the extreme and, if it were used for policy-making and planning, could have led to a cap on the investment of resources for vaccination in the hospital’s catchment area. This means that far more effort should be invested in supporting district health management teams to calculate the catchment population accurately.

Secondly, indicators in general are required to quantify an input, a process, or the output/outcome of an activity. Health indicators should therefore describe the health of the population or the quality of a health facility to monitor and evaluate ongoing development. Some of the indicators in MTUHA seem questionable for this purpose: For example:

○ “Sum of deliveries performed at the facility or by birth attendants/ total number of < 1 year olds per year”.

By including deliveries carried out by traditional birth attendants outside the hospital this indicator becomes unusable for describing the quality of obstetric care in the hospital’s maternity department. It also focuses upon the accessibility of obstetric care, which is not under the facility’s direct influence.

Thirdly, the fact that data collected for indicators in MTUHA book 2 is not included in any of the reporting forms is clearly a design fault. It is also a source of inefficiency because it means that this information does not get passed onto the next level and therefore cannot be made available for decision-making.

Well functioning feedback mechanisms are essential for generating improvements in ongoing processes. This assessment showed there is practically no feedback within MTUHA, neither within the hospital nor from a superior level. Communication between the different stakeholders was reduced to the minimum of report submission and receipt. In this particular case feedback from the district level was insufficient. Only the lateness of reports generated a reaction – presumably, so that reporting to the next level could be completed. However, the quality of the reports appeared to go unnoticed. Even though 8 of the 11 forms comprising the hospital report were incomplete and 3 were not filled out at all no complaint was ever received. The lack of feedback with regard to the data collection and analysis may have been linked to the poor understanding of the data in general. A simplification of the data collection and analysis, namely the indicators might lead to an improvement of the feedback process.

Hospital staff were found to be aware that OPD cases were routinely underreported in the MTUHA register 5. The total of OPD cases in the hospital report is far higher than the total of the registered cases in MTUHA book 5. The hospital report total matches the staff’s clinical experience. This suggests that the evidently wrong data was adapted accordingly. However, the basis of epidemiology lies in the power of a trusted ‘data-set’ to change the ‘mind-set’ [23]. Such examples of a data-set’ being adapted to fit the ‘mind-set’ render data collection and analysis meaningless.

Finally, a clear difference should be made depending on whether the data itself is not available or the actual service. By clearly indicating which services are not provided at the hospital a greater awareness of the actual situation there could be created.

### Data use

MTUHA generates a multitude of indicators and absolute numbers which are valuable for an evaluation of hospital level activities. Table 4 shows the poor alignment between MTUHA data and the hospital development plan. The hospital’s successful alignment of the HIV/AIDS data in the plan to the NACP database and the resulting reduction of workload is an encouraging example. It seems feasible to similarly adapt other health indicators required by the hospital development plan to those supplied by MTUHA. This would also have the benefit of bringing greater meaning to the use of MTUHA at the hospital.

The smooth implementation of the NACP data base at the CTC raises the question as to how this had been achieved. Reference was made to extensive training as well as to staff incentives. It is possible that this reflects the considerable resources that have been designated to the response to HIV/AIDS. The question must however be asked why the collection of HIV/AIDS data was not integrated into MTUHA. In such a setting it is likely that when resources are concentrated on a particular area of data collection, then a diluting effect will be experienced in others areas.

## Conclusions/recommendations

There is a need to address internal inconsistencies between and within the different types of data tools in MTUHA. This process of revision could also include a deletion of repeated indicators and an updating of the MTUHA classification of diseases. Technical assistance from higher levels could be provided to tackle the difficulties associated with calculating population based indicators at district level. Then it would be possible for a useable figure to be supplied to the facilities.

Regarding the extensiveness of MTUHA an argument could be made for the managerial aspects to be removed from the HIS and for the emphasis to be placed firmly on data collection for routine services. Alternatively, the managerial aspects could be sharpened for each type of facility, with a particular focus upon the hospital level. Facility management has been found wanting in Tanzania [24] – a potential, partial solution would be a tailor-made training and mentoring for hospital managers. This could include familiarisation and explanation of the broader managerial aspects of MTUHA. A template for hospital development plans that makes use of MTUHA indicators could also be usefully introduced.

Facility based training on MTUHA, including for non-clinical staff, could be initiated by an appropriately sensitised management. In particular, issues of concern to staff such as how to classify return visits, or common errors such as how to count the length of patient stay could be clarified in such a forum. Staff with particular difficulties regarding mathematics could hereby be identified. Overall, there is a need to raise the status of data collection. The attention it receives during supportive supervision from those from the next level should also be increased.

## Abbreviations

CTC: Care and treatment clinic; DHMT: District health management team; EPI: Expanded program for immunizations; HIMS: Health information management system; HIS: Health information system; MTUHA: **M**fumo wa **T**aarifa za **U**endeshaji **H**uduma za **A**fya; NACP: National AIDS Control Program; OP: Out patient; OPD: Out patient department; PMTCT: Prevention of mother to child transmission; PICT: Provider initiated counselling and testing; RMO: Regional medical office; WHO: World Health Organisation.

## Competing interests

All authors declare that they have no competing interests.

## Authors’ contributions

MW undertook the literature review, conceived the methods, developed the interview guideline, gained ethical clearance, collected the data, conducted the analysis. OM supported the data collection and analysis. HP assisted with the literature review and analysis. Contributed to all stages of manuscript preparation including submission. PH contributed to the study design, supervised the field work, contributed to the discussion. RK contributed to the study design, supervised the study, revised the later stages of the manuscript. All authors read and approved the final version.

## Pre-publication history

The pre-publication history for this paper can be accessed here:

http://www.biomedcentral.com/1472-6963/14/91/prepub
